# Year in review 2013: *Critical Care* – sepsis

**DOI:** 10.1186/s13054-014-0578-x

**Published:** 2014-10-15

**Authors:** Etienne de Montmollin, Djillali Annane

**Affiliations:** Service de Réanimation Polyvalente, Hôpital Raymond Poincaré, 104 bd Raymond Poincaré, 92380 Garches, France

## Abstract

This review presents key publications from the research field of sepsis published in *Critical Care* and other relevant journals during 2013. The results of these experimental studies and clinical trials are discussed in the context of current scientific and clinical background. The discussion highlights and summarises articles on four main topics: sepsis pathogenesis, diagnostic and prognostic biomarkers, potential new therapies, and epidemiologic and outcome studies.

## Introduction

Despite intense experimental and clinical research activity over the past decades, sepsis still remains an elusive syndrome. Actual understanding has led to international recommendations on diagnosis and treatment [1], but management of severe sepsis and septic shock in the ICU still represents a major challenge for clinicians in 2014, with high mortality rates. The past year’s contribution to the field of sepsis research was quite prolific, and the aim of this review is to summarise the relevant findings of research articles that were published in 2013 in *Critical Care* and other relevant journals. We focus on advances in the understanding of sepsis physiopathology, diagnostic and prognostic biomarkers, potential new therapies, and epidemiologic and outcome studies.

## Sepsis pathophysiology

In pulmonary infection by *Staphylococcus aureus*, host defence mechanisms contribute to lung damage by releasing damage-associated molecular patterns. High-mobility group box 1 (HMGB1) is a damage-associated molecular pattern of particular interest, acting as a cytokine via the toll-like receptor 4 (TLR4) and the receptor for advanced glycation end-products (RAGE). In a mouse model of *S. aureus* pneumonia, histologic signs of acute lung injury were reduced by adjunction of anti-HMGB1 antibodies as well as levels of the cytokine IL-1β [2]. Using TLR4 and RAGE knockout mice in the same septic model, the authors showed that TLR4 deficiency did not influence lung pathology but RAGE deficiency led to attenuated lung damage. RAGE-deficient mice had lower tumour necrosis factor alpha and IL-6 levels in bronchoalveolar fluid at 24 hours post-infection, but not TLR4-deficient mice. This study suggests distinct harmful roles of HMGB1 and RAGE, but not of TLR4, in the development of lung injury during the early phase of severe pneumonia caused by *S. aureus*. These findings are in accordance with the association found between elevated levels of soluble RAGE and mortality and acute respiratory distress syndrome (ARDS) in 33 patients admitted to the ICU for community-acquired pneumonia [3].

B- and T-lymphocyte attenuator (BTLA) is a co-inhibitory receptor that is known to potently inhibit CD4^+^ T-cell and B-cell function [4]. Whether BTLA plays a role in driving lymphocyte dysfunction and apoptosis is questioned. In 11 septic ICU patients, a significantly higher percentage of circulating lymphocytes expressing BTLA was found, compared with patients with nonseptic systemic inflammatory response syndrome (SIRS) [5]. Furthermore, a higher frequency of CD4^+^BTLA^+^ cells was found in SIRS patients who subsequently developed infection. In an experimental caecal ligation and puncture mouse sepsis model, including BTLA^−/−^ mice, it was shown that BTLA contributed to the apoptosis of T cells and B cells in the thymus and the spleen, and was associated with peripheral T-cell and B-cell reduction. BTLA could be a potential therapeutic target/risk marker for patients susceptible to developing subsequent secondary infections.

Nuclear factor-κB is a key transcriptional regulator of inflammation and organ injury. In this respect, Devaney and colleagues investigated the role of nuclear factor-κB inhibition by intrapulmonary delivery of a viral vector encoding the nuclear factor-κB inhibitor IκBα in a rat model of *Escherichia coli* pneumonia [6]. In acute pneumonia (that is, 4 hours after bacterial instillation), IκBα overexpression enhanced animal survival, improved arterial oxygenation, static lung compliance and pulmonary permeability, and significantly decreased alveolar IL-1β production. However, in a prolonged pneumonia model (that is, 72 hours after bacterial instillation), IκBα overexpression increased the lung *E. coli* bacterial load and the proportion of neutrophils in the alveolar infiltrate, and worsened *E. coli*-induced histologic injury. Alveolar levels of IL-1β and tumour necrosis factor alpha were also increased. This study demonstrates the dual role of nuclear factor-κB in infection, and stresses the detrimental effects of a strategy to inhibit nuclear factor-κB if not temporally targeted.

The innate and adaptive immune systems are involved in the pathogenesis of sepsis, and B cells are thought to contribute to the immunosuppressive shift observed during sepsis. Monserrat and colleagues demonstrated in 52 patients with septic shock a reduction of circulating B lymphocytes and a relation between a low percentage of activated regulatory cells and an increased 28-day mortality [7]. Further studies are warranted to specify the mechanisms by which these alterations contribute to the evolution from sepsis to septic shock.

The signalling molecule nitric oxide may also play a role in the pathophysiology of ARDS. Citrulline is a substrate for nitric oxide synthase. It was hypothesised that citrulline levels would decrease during sepsis, leading to a fall in nitric oxide production in the lung and potentiating the development of ARDS. Ware and colleagues demonstrated that very low citrulline levels are associated with ARDS [8]. This association may be due to a decrease in citrulline availability or citrulline overconsumption for nitric oxide synthesis but also may result from an arginine deficiency syndrome. In any case, the results from this study reinforce the rationale for a study of citrulline supplementation in critically ill patients.

Mitochondrial dysfunction has been implicated as a causative mechanism for reduced activity of immune cells in sepsis. Evolution of mitochondrial respiration function in human peripheral blood immune cells was assessed by Sjövall and colleagues within 48 hours of sepsis onset, and at days 3 to 4 and days 6 to 7, as well as its relation to outcome [9]. Peripheral blood immune cells from 20 patients with severe sepsis or septic shock were analysed with high-resolution respirometry and compared with 31 controls. The results showed that cellular respiration of peripheral blood immune cells presents an amplified capacity during the first week of sepsis, because of an increase in both mitochondrial content and oxidative phosphorylation capacity. Nonsurvivors displayed the same increase in respiration as the survivors. These findings argue against mitochondrial dysfunction in immune cells at sepsis onset.

Malondialdehyde (MDA), an end-product of lipid peroxidation, is one of the molecules involved in toxic effects of oxidative stress. Toufekoula and colleagues demonstrated that MDA levels measured in 93 patients with ventilator-acquired pneumonia due to multidrug-resistant bacteria were increased in cases of hepatic dysfunction or ARDS, but were lower in patients with acute renal failure [10]. Results of this study are important because they confirm that oxidative stress is compartmentalised during sepsis. This compartmentalisation should be borne in mind for designing future trials evaluating antioxidant agents in sepsis.

Septic patients frequently suffer from compromised sensitivity of the vasculature to pressor hormones, such as angiotensin II [11]. Arap1 is a receptor-associated protein of the angiotensin receptor 1 that enhances membrane trafficking leading to improved sensitivity. In an endotoxaemic mouse model, Arap1 was markedly downregulated by sepsis, and sepsis-induced circulatory failure worsened in Arap1-deficient mice [12]. Loss of Arap1 is thus a newly identified mechanism for vascular hyporeactivity in septic patients, and adds to the already identified factors: endothelial injury, arginine–vasopressin system dysfunction, release of other vasodilatory inflammatory mediators, and muscle hyperpolarisation [13].

Vassiliou and colleagues demonstrated that Aquaporin-1, a water channel protein present in migratory cells, was upregulated in mice polymorphonuclear granulocytes after stimulation by lipopolysaccharide (LPS), was involved in LPS-induced polymorphonuclear granulocyte plasma membrane permeability, and may play a role in polymorphonuclear granulocyte migration to the site of infection [14]. Further studies are needed to clarify the regulation of the aquaporin-1 signal transduction pathway and its functional significance to better define its role in sepsis physiopathology.

## Diagnostic and prognostic markers in sepsis

Although the sepsis definition is based on internationally accepted criteria [1], symptoms and signs are highly variable, making clinical recognition and severity assessment very challenging. Sepsis is a time-sensitive emergency, and therefore early diagnostic markers are of paramount importance to improve outcomes. Furthermore, prognostic markers may help improve triage and patient management. Table 1 summarises the diagnostic and prognostic biomarkers review in this section.

**Table 1:** **Summary of diagnostic and prognostic performance of cited biomarkers**

**Biomarker**	**Context**	**Performance**	**Reference**
**Diagnostic markers**			
PSP, sCD25, and HBP within 6 hours of admission	Prospective monocentric; 219 patients admitted to the ICU	Diagnosis of sepsis versus SIRS: PSP (cutoff value 30 ng/ml), Se 90% and Sp 83%; sCD25 (cutoff value 2.5 ng/ml), Se 83% and Sp 83%; HBP (cutoff value 50 ng/ml), Se 78% and Sp 36%	[20]
sCD25, IL-10, and IFNγ on days 1 and 7 of hospital admission	Prospective monocentric; 52 patients with SIRS criteria	Diagnosis of bacteriaemic SIRS (day 1): sCD25 (cutoff value 19.9 ng/ml), Se 83% and Sp 83%; IL-10 (cutoff value 3.05 pg/ml), Se 88% and Sp 75%; IFNγ (cutoff value 9 pg/ml), Se 45% and Sp 70%	[21]
Presepsin at admission	Prospective monocentric; 859 ED patients with SIRS criteria	Presepsin >317 pg/ml: diagnosis of sepsis, Se 71% and Sp 86%	[16]
Presepsin at admission	Prospective bicentric; 186 ED patients with SIRS criteria	Presepsin >600 pg/ml: diagnosis of sepsis, Se 79% and Sp 62%	[17]
**Prognostic markers**			
Red blood cell distribution width at admission and at 72 hours	Prospective monocentric; 329 ED patients, severe sepsis or septic shock	Increased baseline RDW and ΔRDW at 72 hours after admission >0.2%: 28-day mortality, OR =9.97 (95% CI =1.99 to 49.91); hospital length of stay, OR =25.45 (95% CI =14.69 to 36.21	[30]
PCT change at 72 hours from baseline	Retrospective multicentre; 154 ED patients with sepsis	Per 10% PCT increase from baseline: ICU mortality, OR =1.3 (95% CI =1.1 to 1.15); 80% PCT decrease from baseline: ICU mortality, Se 91% and NPV 90%	[22]
sCD25 and IL-10 at days 1 and 7 of hospital admission	Prospective monocentric; 52 patients admitted to the hospital with SIRS	ICU mortality of bacteriaemic patients with SIRS: day 1 sCD25 > 19.9 ng/ml, OR =1.12 (95% CI =1.01 to 1.25); day 1 IL-10 > 3.05 pg/ml, OR =1.86 (95% CI =0.98 to 3.52)	[21]
Presepsin at admission	Prospective monocentric; 859 ED patients with SIRS criteria	Evolution to severe sepsis (cutoff value 449 pg/ml), Se 82% and Sp 72%; evolution to septic shock (cutoff value 550 pg/ml), Se 86% and Sp 64%; 28-day mortality (cutoff value 556 pg/ml), Se 62% and Sp 67%	[16]
Serum MDA at day 1	Prospective multicentre; 328 patients with severe sepsis	MDA >4.11 nmol/l: 30-day mortality, HR =1.05 (95% CI =1.02 to 1.09); 6-month mortality, HR =1.05 (95% CI =1.02 to 1.09)	[36]
372 T/C polymorphism of TIMP-1	Prospective multicentre; 275 patients with severe sepsis	If 372 T/C polymorphism present: 30-day mortality, OR =2.08 (95% CI =1.06 to 4.09)	[37]
Kallistatin at days 1 and 4	Prospective monocentric; 54 ICU patients with severe CAP	Kallistatin >8.3 μg/ml: ICU mortality, OR =0.11 (95% CI =0.01 to 1.06)	[18]
Ang-1/Ang-2 ratio at fever onset	Prospective bicentric; 99 cancer and chemotherapy-induced febrile neutropaenia patients	Ang-1/Ang-2 ratio >5: development of septic shock, RR =5.47 (95% CI =1.93 to 15.53); 28-day mortality, RR =4.20 (95% CI =1.60 to 11.05)	[28]

Ang, angiopoietin; CAP, community-acquired pneumonia; CI, confidence interval; ED, emergency department; HBP, heparin binding protein; HR, hazard ratio; IFNγ, interferon gamma; IL, interleukin; MDA, malondialdehyde; NPV, negative predictive value; OR, odds ratio; PCT, procalcitonin; PSP, pancreatic stone protein; RDW, red blood cell distribution width; RR, relative risk; sCD25, soluble CD25; Se, sensitivity; SIRS, systemic inflammatory response syndrome; Sp, specificity; TIMP-1,tissue inhibitor of matrix metalloproteinase 1.

Presepsin is a subtype of soluble CD14, which is a receptor for LPS and LPS binding protein complexes expressed on macrophage, monocyte, and granulocyte cells. Previous clinical studies have found that plasma presepsin levels were increased in sepsis and correlated with sepsis severity [15]. Two different studies published in *Critical Care* have evaluated this new potential diagnostic and prognostic biomarker in patients admitted to the emergency department with SIRS criteria. The diagnostic accuracy for sepsis of presepsin found by Liu and colleagues was correct with a sensitivity of 71% and a specificity of 86% for a cutoff value of 317 pg/ml. Presepsin was found to be an independent predictor of severe sepsis and septic shock compared with septic patients, and was an independent predictor of 28-day mortality [16]. In the second study by Ulla and colleagues, the sensitivity and specificity of presepsin for a cutoff value of 600 pg/ml were, respectively, 79% and 62%. Presepsin values at admission to the emergency department correlated with 60-day in-hospital mortality in patients with severe sepsis and septic shock [17]. These studies indicate that presepsin could be a promising tool for diagnosing sepsis in the emergency department, and appears superior to procalcitonin (PCT) for risk stratification and evaluation of prognosis.

Lin and colleagues evaluated kallistatin levels in patients with severe community-acquired pneumonia [18]. Day 1 kallistatin plasma levels were lower in patients who had septic shock and developed ARDS, and the sensitivity and specificity for predicting death for a cutoff value of 6.5 μg/ml were, respectively, 81% and 54%. These findings indicate that kallistatin may be protective against severe community-acquired pneumonia, which implies possible therapeutic benefits of kallistatin in these patients. Indeed, in a recent experimental study of a mouse polymicrobial sepsis model, administration of kallistatin reduced systemic inflammation and endothelial activation, and improved survival [19].

In 219 unselected patients admitted to an ICU, three diagnostic biomarkers were evaluated and compared with PCT: pancreatic stone protein, a lectin-binding protein; soluble CD25 (sCD25), which is the soluble form of the IL-2 receptor alpha chain; and heparin binding protein, an inflammatory mediator contained in neutrophil secretory granules [20]. All biomarkers were measured in plasma collected within 6 hours of admission. Levels of PCT, pancreatic stone protein, and sCD25, but not of heparin binding protein, were significantly higher in septic patients than in noninfectious SIRS patients. Using receiver operating characteristic analysis, the optimal cutoff values were 30 ng/ml (sensitivity 90%, specificity 83%) for pancreatic stone protein and 2.5 ng/ml (sensitivity 83%, specificity 83%) for sCD25. In comparison, PCT with a cutoff value of 1 ng/ml had a sensitivity of 71% and specificity of 82%. In this study, pancreatic stone protein and sCD25 performed at least as well as PCT for sepsis diagnosis in SIRS patients, and warrant further assessment for clinical decision making.

In a prospective study of 52 patients admitted with SIRS, diagnostic performance for bacteraemia at day 1 of IL-10 and sCD25 showed respectively sensitivity/specificity of 78%/80% and 87%/75% [21]. In multivariate analysis, IL-10 and sCD25 at day 1 were independent predictors of mortality (respectively odds ratio (OR) =1.86 and OR =1.12, *P* <0.05). In conclusion, sCD25 and IL-10 are early diagnostic and prognostic markers but lack sensitivity and cannot be recommended for routine use at the moment.

In a US-based retrospective observational multicentre study, the prognostic information obtainable from a change of PCT at 72 hours from baseline value was investigated in a cohort of 154 patients admitted to the ICU with a diagnosis of sepsis [22]. A PCT increase between baseline and 72 hours after ICU admission was associated with increased ICU mortality (OR per 10% PCT increase =1.3, 95% confidence interval (CI) =1.1 to 1.5, *P* =0.001). With a cutoff value of 80% PCT decrease at 72 hours, the sensitivity and the negative predictive value for ICU mortality were excellent, respectively 91% and 90%. These data suggest that a PCT decrease >80% at 72 hours after ICU admission may help to identify patients with reduced mortality risk, and prompt early ICU discharge.

Downregulation of monocyte human leukocyte antigen DR (HLA-DR) surface expression measured by flow cytometry has been identified as a biomarker of sepsis-induced immunosuppression [23]. Flow cytometry can be difficult to perform due to specific laboratory requirements. Cajander and colleagues demonstrated that the mRNA expression level of HLA-DR monitored by quantitative real-time polymerase chain reaction correlates highly with surface expression of HLA-DR measured by flow cytometry and could become a routine technique in this context [24]. Immunoparalysis is also characterised by defective B lymphocytes and low immunoglobulin production, including immunoglobulin M (IgM) that is crucial for the opsonisation and clearance of invading microorganisms. Giamarellos-Bourboulis and colleagues demonstrated in 332 critically ill patients with SIRS that IgM levels in patients with severe sepsis progressing to septic shock remained stable in nonsurvivors, whereas IgM of survivors presented an early peak with a subsequent gradual decrease [25]. However, there are to date insufficient data to use IgM level kinetics as prognostic markers. Of note, the use of IgM-enriched immunoglobulin preparation in a recent trial of 33 septic shock patients failed to improve mortality as compared with standard therapy [26].

Angiopoietin (Ang)-1 and Ang-2 play a critical role for endothelial barrier integrity. The Ang-2/Ang-1 ratio has emerged as a promising endothelial-associated sepsis biomarker [27]. In a prospective study of 99 patients with cancer and chemotherapy-induced febrile neutropaenia, Luz Fiusa and colleagues evaluated the serum Ang-2/Ang-1 ratio at fever onset as a prognostic biomarker [28]. The Ang-2/Ang-1 ratio was much higher in patients who developed septic shock compared with patients with noncomplicated febrile neutropaenia. For an Ang-2/Ang-1 ratio >5, the relative risk of developing septic shock was 5.47 (95% CI =1.93 to 15.53, *P* =0.001). The 28-day mortality risk for a ratio >5 was 4.20 (95% CI =1.60 to 11.05, *P* =0.004), confirming that the Ang-2/Ang-1 ratio is a biomarker of septic shock development and poor outcome for febrile neutropaenia patients. As the severity of microvascular alterations is strongly associated with organ dysfunction and mortality [29], the Ang-2/Ang-1 ratio appears to be of particular interest. At this stage, however, therapies to specifically target the microcirculation are still being investigated.

The red blood cell distribution width represents an index of the heterogeneity of erythrocytes and has been shown to have predictive value for all-cause mortality in critically ill patients, although the mechanism of the association remains unclear. Kim and colleagues demonstrated in 329 severe sepsis and septic shock patients that the combination of both an increased red blood cell distribution width at baseline and an increase in red blood cell distribution width from baseline during the first 72 hours after admission was an independent predictor of 28-day mortality (OR =9.97, 95% CI =1.99 to 49.91, *P* =0.005) [30]. These results are in accordance with those of Sadaka and colleagues, who showed that the red blood cell distribution width at day 1 in septic shock patients fared better than either the Acute Physiology and Chronic Health Evaluation II or Sequential Organ Failure Assessment score as a prognostic marker [31].

The Japanese Association for Acute Medicine Disseminated Intravascular Coagulation scoring system includes SIRS criteria, platelet count, fibrinogen level, and prothrombin time [32]. Gando and colleagues showed that disseminated intravascular coagulation-positive patients with severe sepsis at admission exhibited higher prevalence of septic shock and positive blood cultures [33]. The 28-day mortality gradually increased with the Japanese Association for Acute Medicine Disseminated Intravascular Coagulation score, and the score at day 1 was an independent predictor of 28-day mortality.

Abnormal body temperature is a frequent finding in patients with sepsis, and it was hypothesised that temperature patterns could be early indicators of sepsis and prognostic markers. In a retrospective case–control study, Drewry and colleagues identified abnormal temperature patterns 72 hours prior to clinical suspicion of sepsis (described as an ‘increase in amplitude, change in frequency or loss of variability’) in afebrile ICU patients as predictive of subsequent diagnosis of sepsis (OR =4.43, 95% CI =1.31 to 15.00, *P* =0.017) [34]. In a second prospective multicentre observational study of 624 patients admitted to the ICU with severe sepsis, Kushimoto and colleagues demonstrated that hypothermia during the first 24 hours of diagnosis, defined as body temperature ≤36.5°C, was identified as an independent predictor of 28-day mortality in patients with severe sepsis (OR =1.95, 95% CI =1.25 to 3.04, *P* =0.003) [35]. Elevated body temperature, in contrast, was not associated with an increased disease severity or risk of mortality. These findings are of importance, as temperature is easily recorded at the bedside and could bring useful diagnostic and prognostic information. However, care should be taken regarding how and when body temperature is recorded, which should be standardised in future prospective studies.

In a prospective multicentre observational study of 328 patients diagnosed with severe sepsis, Lorente and colleagues demonstrated that serum MDA levels at days 1, 4, and 8 were significantly higher in nonsurviving septic patients compared with survivors [36]. For a cutoff value of 4.11 nmol/l, the sensitivity and specificity to predict mortality were only respectively 42% and 82%. Overall, although high MDA levels are sustained throughout the first week of sepsis, the clinical utility for predicting patient outcome is insufficient.

The 372 T/C polymorphism of tissue inhibitor of matrix metalloproteinase-1 (TIMP-1) is the most studied genetic variant of this regulator of matrix metalloproteinases, involved in leukocyte recruitment and modulation of inflammatory and prothrombotic response in sepsis [37]. Lorente and colleagues showed that the 372 T/C genetic polymorphism of TIMP-1 was associated with higher serum levels of TIMP-1 and higher 30-day mortality (OR =2.08, 95% CI =1.06 to 4.09, *P* =0.03) [38]. Determination of the 372 T/C genetic polymorphism of TIMP-1 has prognostic implications and could help in the selection of patients who may benefit from modulation of the matrix metalloproteinase/TIMP balance.

## Potential new therapies

### Experimental studies

The immunosuppressive state of late sepsis makes patients more at risk of secondary nosocomial infections, amongst them fungal infections. Programmed cell death-1 and cytotoxic T-lymphocyte antigen 4 are negative co-stimulatory molecules that suppress T-cell function. In an experimental study of a mouse model of primary candidaemia and a two-hit model of caecal ligation and puncture followed by candidaemia, antibody blockade of either programmed cell death-1, programmed cell death-1 ligand, or cytotoxic T-lymphocyte antigen 4 significantly improved survival [39]. This survival improvement was associated with increased production of interferon gamma and reversion of the fungal-induced depression of HLA-DR expression on monocytes and dendritic cells. Furthermore, in an *in vitro* study on blood from septic critically ill patients, blockade of the programmed cell death-1–programmed cell death-1 ligand pathway decreased apoptosis and improved immune cell function [40]. These data support the concept that immune-adjuvant therapy could improve the treatment of fungal infections.

Glycyrrhizin is a glycoside compound extracted from the plant *Glycyrrhiza glabra*, and has shown anti-inflammatory properties via binding to HMGB-1 and inhibition of this late sepsis proinflammatory cytokine [41]. Wang and colleagues demonstrated in a porcine endotoxaemic model that glycyrrhizin improved systemic haemodynamics and enhanced pulmonary oxygen exchange [42]. Serum proinflammatory cytokine levels were reduced, and infiltration by inflammatory cells was significantly reduced in the lung, liver, and kidney tissues. Thus, through modulation of the inflammatory response and attenuation of organ damage, glycyrrhizin should be seen as a potential agent in the treatment of sepsis and requires additional experimental and clinical investigation.

Antimicrobial peptides are proteins able to bind and neutralise LPS, and may kill bacteria without releasing proinflammatory factors [43]. In an experimental study of the mouse caecal ligation and puncture septic model, effects of a newly synthetised antimicrobial peptide, coined Pep 2.5, were assessed [44]. The authors demonstrated that continuous infusion of Pep 2.5 reduced circulating proinflammatory mediator levels (IL-6, IL-10, and MCP-1) compared with the sepsis control group, and that Pep 2.5 reduced CD14 (contributor to the TLR4-mediated LPS recognition) mRNA tissue expression in the heart, lung, and spleen compared with controls. This antimicrobial peptide may thus have the potential for further development as an anti-infective treatment in sepsis.

### Human studies

Rimmelé and colleagues showed that the use of haemoadsorption devices on the blood of septic shock patients enabled the capture of monocytes and neutrophils, but not lymphocytes, and led to a local release of IL-8 and changes in T-cell function [45]. This more systematic approach with tight immune monitoring is important to better understand the action of haemoadsorption devices in sepsis.

Thymosin alpha 1 (Tα1) is a thymic peptide that has immunomodulating effects primarily by affecting the enhancement of T-cell functions [46]. In a multicentre randomised control trial including 361 patients with severe sepsis admitted to the ICU, Wu and colleagues showed that administration of Tα1 for 5 days after sepsis diagnosis significantly reduced in-hospital mortality compared with placebo (relative risk of death 0.73, 95% CI =0.54 to 0.98, *P* =0.032), but with no significant difference in ICU mortality, length of ICU stay, and duration of mechanical ventilation [47]. Of note, greater improvement in mHLA-DR was observed in the Tα1 group at days 3 and 5, suggesting improved immune function in this group compared with placebo. This study, the first and only one conducted in patients with severe sepsis, demonstrates that Tα1 therapy could reduce mortality, and that larger multicentre trials are warranted to confirm these findings.

Analysing human studies of potential new therapies in sepsis, it is important to acknowledge that the overall mortality associated with septic shock has now decreased to 18% in the most recent trials [48,49]. This improvement results from the implementation of international guidelines [1] and generalisation of interventions such as early administration of appropriate antibiotics, fluid resuscitation, and so forth. The challenge to demonstrate a mortality benefit with new therapies will now be greater given the lower baseline mortality. In this respect, the study by Wu and colleagues demonstrating a reduction in hospital mortality after administration of the immunomodulating agent Tα1 is of particular interest.

## Outcome studies

Many patients surviving sepsis will develop complications associated with significant long-term sequelae that will impact on health-related quality of life. Previous follow-up studies of sepsis patients with evaluation of quality of life did not exceed 3 years, and thus Cuthbertson and colleagues conducted a prospective cohort study on 439 patients with severe sepsis with a quality of life follow-up (with Short Form-36 and euroQOL-5D questionnaires) at 3.5 and 5 years [50]. Patients had an ongoing high mortality after severe sepsis with survival rates of 57% in hospital, 42% at 3.5 years, and 39% at 5 years. Furthermore, patients had significantly lower physical quality of life compared with the population norm, but only slightly reduced mental quality-of-life scores. These data, similar to other critically ill cohorts, give invaluable insights for the improvement of long-term post-ICU care [51] and when evaluating cost-effectiveness of care in severe sepsis patients.

In a prospective observational study of 1,001 patients admitted to the ICU for severe sepsis between 2004 and 2009, Phua and colleagues showed that 41.5% of patients had negative culture [52]. Culture-negative patients had fewer comorbid conditions and lower Acute Physiology and Chronic Health Evaluation II and Sequential Organ Failure Assessment scores, and the lungs were the most frequent site of infection. While providing useful data on culture-negative sepsis, this study cannot conclude on the very nature of culture-negative sepsis, and further studies are warranted on this topic.

Sakr and colleagues investigated the influence of gender on the epidemiology of severe sepsis in ICU patients, and its possible impact on mortality [53], and showed that prevalence of severe sepsis was lower in female patients but female gender was independently associated with a higher risk of in-ICU death in patients with severe sepsis (OR =2.33, 95% CI =1.23 to 4.39, *P* =0.009). These epidemiological findings should lead to further investigations of the relation between immune response to sepsis and sex steroid hormones.

## Conclusion

This year in review has reviewed articles published in *Critical Care* and other relevant journals in 2013. Much work has been done to better understand the pathophysiology of sepsis, and advances have been made concerning the immune and inflammatory response, oxidative stress, and mitochondrial dysfunction. Several studies concerned the evaluation of diagnostic and prognostic biomarkers. Some of these biomarkers seem promising, and future research in the sepsis field should emphasise these markers because early recognition of severe sepsis or septic shock is paramount to improve patient survival. In this respect, new rapid identification of causative pathogens by techniques such as mass spectrometry and real-time polymerase chain reaction is also a major evolution for patient care. The observed reduction in mortality from patients with sepsis in the past decade has come mainly from the improvement of supportive care. Further improvement in outcome will come from identification of new therapeutic targets via basic and clinical research.

## Note

This article is part of a collection of Year in review articles in *Critical Care*. Other articles in this series can be found at http://ccforum.com/series/Yearinreview2013

## Abbreviations

Ang: Angiopoietin
ARDS: Acute respiratory distress syndrome
BTLA: B- and T-lymphocyte attenuator
CI: Confidence interval
HLA-DR: Human leukocyte antigen DR
HMGB1: High-mobility group box 1
IgM: Immunoglobulin M
IL: Interleukin
LPS: Lipopolysaccharide
MDA: Malondialdehyde
OR: Odds ratio
PCT: Procalcitonin
RAGE: Receptor for advanced glycation end-products
sCD25: Soluble CD25
SIRS: Systemic inflammatory response syndrome
TIMP-1: Tissue inhibitor of matrix metalloproteinase-1
TLR4: Toll-like receptor 4
Tα1: Thymosin alpha 1
